# Dental Caries, Tooth Erosion and Nutritional Habits in a Cohort of Athletes: A Cross-Sectional Study

**DOI:** 10.3390/nu17030543

**Published:** 2025-01-31

**Authors:** Baptiste Mielle, André Júdice, Luís Proença, Vanessa Machado, Ana M. Vieira, José João Mendes, Cristina Manso, Cecília Rozan, João Botelho

**Affiliations:** Egas Moniz Center for Interdisciplinary Research, Egas Moniz School of Health and Science, 2829-511 Almada, Portugalajudice@egasmoniz.edu.pt (A.J.); lproenca@egasmoniz.edu.pt (L.P.); vmachado@egasmoniz.edu.pt (V.M.); jmendes@egasmoniz.edu.pt (J.J.M.); cmanso@egasmoniz.edu.pt (C.M.); jbotelho@egasmoniz.edu.pt (J.B.)

**Keywords:** sports dentistry, sports nutrition, dental erosion, dental caries

## Abstract

Background/Objectives: this study aimed to study the association of dental caries and erosion in athletes with dietary and oral health habits. Methods: An observational cross-sectional study was conducted at the Sports Dentistry department of a university clinic. Intraoral examination included the International Caries Detection and Assessment System (ICDAS II) index and the Basic Erosive Wear Examination (BEWE). A questionnaire was completed with sociodemographic data, and dietary and oral hygiene habits. An inferential and multivariable regression analysis was performed to study the association of dental caries and erosion with confounding variables. Results: A total of 80 athletes were included. The prevalence of dental caries and erosion was 50.0% and 40%, respectively. Significant associations were observed between self-perceived oral health and caries lesions, with “Good” (−5.01, *p* < 0.001) and “Very good” (−5.46, *p* < 0.001) perceptions linked to fewer lesions. BEWE scores revealed significant associations with meal frequency uncertainty (−12.56, *p* = 0.014) and uncertainty about the last dental visit (8.82, *p* = 0.014). Self-perceived oral health as “Good” or “Very good” was associated with lower dental erosion (*p* < 0.010). Other demographic and behavioral factors were not significantly associated with caries or erosion. Conclusions: this cohort of athletes exhibited a high prevalence of dental caries and erosion, with associated dietary and oral hygiene habits. These results highlight the need for targeted dietary counseling and oral health education for athletes, whose nutritional needs may lead to increased consumption of sugar-rich diets.

## 1. Introduction

Regular physical activity is widely acknowledged as vital for a healthy lifestyle across all ages [1]. Recently, global interest in sports for well-being has surged, with more people participating in physical activities, both amateur and professional. This aligns with advancements in sports medicine, a multidisciplinary field aimed at optimizing athletic performance and health. Within this field, sports dentistry is becoming a crucial but often overlooked specialty, addressing athletes’ unique oral health challenges [2,3].

Athletes are highly vulnerable to dental caries and erosion, significant concerns in sports medicine [4]. Dental caries involves localized tooth demineralization due to acids produced by bacteria, while erosion is due to non-bacterial acid exposure causing overall irreversible enamel loss [5]. Factors like high-sugar energy drinks, acidic supplements, and intense physical exertion impacting saliva flow and composition exacerbate these phenomena [6]. These oral health conditions may adversely affect an athlete’s ability to do sports and overall well-being.

Sports nutrition is crucial for enhancing athletic performance, affecting energy, endurance and recovery [4,7]. Nutritional strategies for pre-, intra- and post-exercise phases focus on carbohydrates, electrolytes and energy-dense foods or drinks [4,8]. The frequent consumption of such aids, high in fermentable sugars and acids, increases the risk of dental caries and erosion [9]. Additionally, decreased salivary flow during intense training or competition exacerbates this risk, as saliva is essential for neutralizing acids and promoting remineralization. Despite the clear links between nutrition, oral health and athletic performance, the oral health needs of athletes remain underexplored in both clinical practice and research [4]. Understanding the specific risk factors for dental caries and erosion in athletes is critical for developing targeted preventive strategies.

Tooth decay is influenced by diet, oral care, saliva composition, and environmental and behavioral factors. Evaluation methods like the Caries Management by Risk Assessment (CAMBRA) system offer a methodical way to assess susceptibility by examining these factors [10]. The recognition of such risk factors (such as fermentable carbohydrate intake, inadequate oral hygiene, and decreased saliva production) and protective elements (like fluoride usage and salivary buffering ability) inform personalized strategies [10]. Implementing this evidence-based model is crucial when studying groups like athletes, who may be more prone to dental decay due to specific dietary habits and oral health challenges [11,12].

This research adheres to CAMBRA’s principles and seeks to contribute to targeted preventive measures for this high-risk population. This study aims to bridge this gap by investigating the association between dental caries and erosion in athletes, and some dietary- and oral health habits. Our hypothesis is that athletes with a higher consumption of acidic or sugary beverages, and inadequate oral health habits, will exhibit a higher prevalence of dental caries and erosion compared to athletes with healthier dietary and oral hygiene practices.

## 2. Methods

### 2.1. Study Design, Setting and Participants

For the present cross-sectional study, data from a cohort of athletes followed at a Sports Dentistry Department of a university clinic (Egas Moniz Dental Clinic, Almada, Portugal) were used. The data were gathered through the consecutive sampling of new patients seeking initial consultations from September 2023 to March 2024. The Egas Moniz Ethics Committee approved the study (1285/2023, approval date: 30 November 2023), and all participants provided informed consent. This report adheres to the Strengthening the Reporting of Observational Studies in Epidemiology (STROBE) guidelines [13] (Supplementary Table S1).

### 2.2. Eligibility Criteria and Sampling

To be included in this study, participants had to: have 18 years of age or older; being able to read, understand, and sign the informed consent form; have declared that they play sport at any level; and were seeking initial triage at the Sports Dentistry department. Participants were invited to participate voluntarily and anonymously. Given the lack of studies comparing oral conditions with oral health values and quality of life, a minimum sample size was not estimated thus we carried out for a preliminary design, with a consecutive random sample obtained over a 6-month period.

### 2.3. Reliability and Calibration

Prior to the start of the study, the examiner (BM) was trained in the diagnosis of dental caries and erosive lesions through the detailed study of exemplary photographs, in order to improve the skills for identifying caries and erosion lesions and to train the use of the Basic Erosive Wear Examination (BEWE) and International Caries Detection and Assessment System II (ICDAS II) indices. Secondly, 10 individuals were selected to be examined both by the examiner and by an experienced observer (CR), considered to be the Gold Standard. Overall, a very good agreement was achieved for BEWE (85.7%, Standard Error [SE] = 11.1) and ICDAS (82.3%, SE = 12.1).

### 2.4. Variables

#### 2.4.1. Outcome Variables

During the oral examination, participants were positioned in a chair while the examiner utilized a disposable dental mirror, a light source, and cotton rolls for cleaning and drying teeth to conduct the assessment. Using the four-level BEWE [14], we assessed erosive lesions on all permanent teeth surfaces, with the exception of third molars. In each sextant, the most affected surface was documented, and the sum of these scores was computed. This total was then utilized to assign an individual risk level as follows: no risk (BEWE ≤ 2); mild risk (3 < BEWE < 8); moderate (9 < BEWE < 13); high (BEWE ≥ 14) [14].

Caries was assessed using the ICDAS II [15]. We chose ICDAS instead of WHO decayed, missing and filled index due to its more sensitivity in estimating caries prevalence and extent compared to the WHO criteria [16]. As per ICDAS criteria, the sites were recorded by a 0 to 6 scoring system: 0 = sound; 1 = first visual change in enamel; 2 = distinct visual change in enamel; 3 = localized enamel breakdown (without clinical visual signs of dentinal involvement); 4 = Underlying dark shadow from dentin; 5 = Distinct cavity with visible dentin; 6 = Extensive distinct cavity with visible dentin.

Patients with carious lesions and/or erosion received required treatment and/or clinical management.

#### 2.4.2. Exposure Variables

Data were collected through a self-reported questionnaire on sociodemographic characteristics and behavioral aspects. This questionnaire was administered prior to taking a panoramic radiography and the clinical oral observation, which are both part of the protocol for first intake appointments. Overall, the information collected included sex, age, frequency and type of sport, an oral health self-assessment and a nutritional questionnaire.

Oral health self-assessment consisted of several questions: “How often do you brush your teeth each day?”, “What do you use for oral hygiene?”, “How regularly do you visit a dentist”, “Have you ever had or do you have any of the oral health problems listed below?”, “How do you rate your oral health?” (very bad, bad, satisfactory, good, very good, don’t know/refuse to answer). In the frequency and type of sport we inquired the sport practiced, the frequency per week, the level of sport (recreational, competitive amateur and high performance).

Questions on nutrition consisted of several questions about nutrition, including the frequency of eating foods with cariogenic and/or erosive potential and sports: soft drinks, coffee, tea, fruit juices and lemonade, energy drinks, isotonic drinks, isotonic gels, whey protein, ergogens (creatine, caffeine), B-Alkaline, sodium bicarbonate, multivitamins, L-carnitine, Omega-3. The frequency of consumption of the above-mentioned items were defined as follows: never (low frequency); once a week (low frequency); two to four times a week (moderate frequency); four to six times a week (moderate frequency); once a day (high frequency); twice a day (high frequency); more than twice a day (high frequency).

### 2.5. Statistical Analysis

Data were stored and recorded and statistically processed using IBM SPSS statistics software, version 29.0.1.0. Descriptive statistical analysis covered the investigation of measures such as mean, median, variance, standard deviation, minimum value, maximum value and interquartile range (IQR). We performed normality tests to determine the distribution of variables in the dataset. A significance level of 5% was adopted for all inferential analyses. To evaluate the associations between demographic, behavioral, and clinical factors with dental caries and dental erosion, multivariable regression models were employed, adjusting for potential confounders. We conducted four regressions: two linear regression models for the number of dental caries and the BEWE score; and, two logistic regression models for the presence of dental caries (presence of dental caries lesions vs. absence of dental caries lesions) and the risk of BEWE (BEWE ≤ 2 vs. BEWE > 2]. Preliminary analyses used univariate models. A multivariate model was then constructed for the outcome variable CAL ≥ 3 mm. Only variables with *p* ≤ 0.25 in the univariate model were included in the multivariate stepwise procedure. According to each linear regression we used the term “Estimate” referring to either number of dental caries lesions or BEWE score.

## 3. Results

### 3.1. Study Sample and Characteristics

From an initial sample of 83 participants, 3 refused to complete the questionnaires after accepting participating. A final sample of 80 participants was obtained, with an age range (24.2 ± 4.0 years), with a predominance of men (70.0%) (Table 1). Regarding dietary habits, 53.8% consumed 2–3 meals per day, while 45.0% consumed 4–5 meals. Most participants (77.5%) practiced one sport, with a median of four training sessions per week (IQR = 3) and two hours per session (IQR = 0.5). A majority had been practicing for less than 5 years (77.5%), with fewer reporting 5–10 years (22.5%), 10–15 years (30.0%), or more than 15 years (43.8%). Sugary drinks or foods were consumed occasionally by 53.8%, on most days by 36.3%, and daily by 6.3%, while 3.8% reported never consuming them.

Participants demonstrated varied oral hygiene habits (Table 2), with all reporting toothbrush use (100%), followed by flossing (53.8%), mouthwash (41.3%), interdental brush (7.5%), and tongue brushing (11.3%). Fluoride toothpaste was reported by 67.5%. Regarding dental visits, 63.8% attended regularly even without complaints, 30.0% visited only when in pain or with complaints, while 2.5% never visited a dentist, and 3.8% were uncertain. Most participants had their last dental visit within six months (60.0%), with fewer reporting visits within 6–12 months (23.8%), over 12 months ago (13.8%), or uncertainty (2.5%). Oral health impacted training and competition in 15.0% and 12.5% of participants, respectively. Self-assessment of oral health was predominantly “Good” (51.3%) or “Very good” (23.8%), with smaller proportions reporting “Satisfactory” (20.0%) or “Bad” (5.0%). Half of the participants (50.0%) had caries, with a mean of 1.6 lesions (SD = 2.6). The prevalence of erosion was 40.0%, BEWE risk mostly absent (80.0%), with mild (12.5%), moderate (5.0%), or high risk (2.5%) reported less frequently.

The distribution of consumption of different types of drinks was also analyzed (Table 3).

### 3.2. Dental Caries and Associated Factors

In the number of active caries lesions, significant associations were observed with self-perceived oral health and dietary habits (Table 4). “Good” (Estimate: −5.01, *p* < 0.001) or “Very good” (Estimate: −5.46, *p* < 0.001) had significantly association with the number of dental caries compared to those with poorer self-perception, with “Satisfactory” ratings approaching significance (Estimate: −5.21, *p* < 0.001). Similarly, lower frequencies of sugary snack consumption were associated with the number of dental caries lesions. Other factors showed no significant associations. For the dichotomous variable of existing dental caries we did not observe significant associations in the final adjusted model (Supplementary Table S2).

### 3.3. Dental Erosion and Associated Factors

The adjusted multivariable linear regression models for the associations between demographic, behavioral, and clinical factors with dental erosion shows statistically significant results with BEWE as a continuous variable (Table 5) but not with is dichotomous transformation (Supplementary Table S3).

Overall, the final adjusted model revealed significant associations between various behavioral and self-perceived factors and overall BEWE score. Participants who reported uncertainty about their meal frequency (“Don’t know”) had significantly lower dental erosion (Estimate: −12.56, *p* = 0.014), while those uncertain about their last dental visit (“Don’t know”) exhibited significantly higher scores (Estimate: 8.82, *p* = 0.014). Self-perceived oral health status showed a clear trend: participants who rated their oral health as “Good” (Estimate: −5.28, *p* = 0.005) or “Very good” (Estimate: −6.12, *p* = 0.003) had significantly lower dental erosion scores compared to those with poorer self-perception, with “Satisfactory” ratings approaching significance (Estimate: −3.85, *p* = 0.052). Conversely, meal frequency categories (4–5 meals/day) and the timing of the last dental visit (>12 months or 6–12 months) did not yield significant associations with BEWE (*p* > 0.05).

## 4. Discussion

This study explored the prevalence and associated factors of dental caries and erosion among athletes, highlighting the importance of self-perceived oral health, dietary habits and oral hygiene behaviors in influencing these outcomes. Collectively, the prevalence of dental caries and erosion shows that 1 out of two athletes had dental caries lesions and 2 out of five had erosion lesions. Oral health self-awareness and regular dental care were the most significant risk indicators associated with the number of dental caries and the BEWE score, revealing its importance as determinants.

The findings underscore the association of self-perceived oral health and preventive behaviors with dental health management among athletes. The high prevalence of dental caries (50%) and erosion lesions (40%) observed in this population emphasizes the need for targeted oral health interventions tailored to the unique dietary and physical demands of athletes. These results are in line with those reported previously in other studies [12,17,18,19,20,21,22,23,24,25,26,27]. The association between oral health self-awareness and reduced dental erosion highlights the potential benefits of promoting regular dental check-ups and personalized education on protective behaviors. These insights could inform the development of preventive strategies aimed at minimizing the burden of oral diseases in athletes, thereby supporting not only their oral health but also their overall performance and well-being. The prevalence of dental caries in our sample was higher compared to the established 35.0% global prevalence data in 2010 [28], but in line with Azeredo et al. in 2020 with 46.25% [29].

Half of the participants presented with dental caries, with self-perceived oral health emerging as a key determinant. Participants who assessed their oral health as “Good” or “Very good” demonstrated significantly fewer caries lesions compared to those with poorer perceptions, corroborating findings from previous studies that underscore the relevance of self-awareness in oral health. Furthermore, dietary habits played a critical role, with lower frequencies of sugary snack consumption being associated with reduced caries prevalence. These findings reinforce the need for targeted dietary counseling and oral health education among young athletes, whose nutritional demands may predispose them to higher consumption of sugar-rich diets.

In contrast, no significant associations were observed for the presence of caries in the adjusted model, suggesting that other unmeasured factors, such as genetic predisposition or fluoride exposure, might contribute to this outcome. This aligns with previous research indicating the multifactorial nature of dental caries, where behavioral, biological, and environmental factors interact.

Regarding the presence of fluoride in toothpaste, 67.5% of the sample reported using fluoride toothpaste, while 8.8% stated they do not use it, and 23.8% said they do not know or did not respond. More than a third of the population denies its use, highlighting the importance of continuing oral hygiene instructions and prevention at the dentist. The ADA recommends brushing for 2 min twice a day with toothpaste containing a low amount of fluoride (1000–1500 ppm) [30].

Regarding athletes’ diet, concerning the frequency of consumption of sugary drinks, foods, or snacks, most of the sample (53.8%) reported consuming them occasionally, while 36.3% stated they consume them most days. Compared to the studies by Gallagher et al. during 2015–2016 [31], it was found that 28.2% of athletes have a high sugar intake in their regular diet. Therefore, the WHO (World Health Organization) recommends moderate intake of free sugars throughout life and suggests reducing their intake to less than 10.0% of total daily energy intake [32]. As for isotonic gels, we found 31.2% of the sample consumes them, while in the mentioned study, the percentage was 70.3%. These differences may be explained by the fact that in Gallagher’s study, all participants are high-level professional athletes, whereas in our investigation, there are athletes of all levels, from amateur to high performance.

Regarding dental erosion, the study revealed significant associations with behavioral and self-perceived factors. Uncertainty regarding meal frequency was associated with significantly lower dental erosion, whereas uncertainty about the timing of the last dental visit was linked to higher erosion scores. These findings could reflect a lack of structured dietary and healthcare habits among these participants. Notably, participants with “Good” or “Very good” self-perceived oral health had lower BEWE scores, further emphasizing the relationship between self-awareness and oral health outcomes. This suggests that promoting a positive perception of oral health could motivate preventive behaviors and reduce the burden of dental erosion. The prevalence of erosion in adults may range from 4% to 82% [19]. In a systematic review by Nijakowski et al. [33], which included 16 studies on the prevalence of erosion in athletes, demonstrated that approximately half of the athletes studied show signs of erosion. These results are consistent with our research. Regarding location, the most affected sextants were the 4th sextant (32.6%) and the 6th sextant (33.8%), which aligns with findings of a higher prevalence of erosion in the lower first molars [34]. Interestingly, meal frequency and the timing of the last dental visit (>12 months or 6–12 months) were not significantly associated with BEWE scores. This highlights the complex interplay of behavioral and clinical factors in dental erosion and underscores the need for further research to unravel these associations.

### Strengths and Limitations

We assessed a self-assessment questionnaire, where responses may be overstated, and participants might feel reluctant to disclose personal details. This limitation underscores the challenge of ensuring complete honesty in self-assessment surveys. Furthermore, additional studies could be conducted to further explore potential risk indicators and specific preventive interventions aimed at reducing the prevalence of dental caries and erosion among athletes, thereby enhancing their overall well-being and sports performance. To achieve more significant results, increasing the sample size would be beneficial, preferably focusing on a more specific sports population, such as conducting the study within a single sport discipline. In the future, it would also be interesting to investigate salivary content before, during, and after training sessions, as suggested by previously [35,36,37]. Furthermore, we intend to prospectively evaluate athletes’ dietary habits, oral health status, and their associated impact on training and performance, with the aim of exploring causal relationships and providing more precise information for future interventions. Additionally, the sample may have limited external validity if athletes with existing oral health concerns were more likely to participate, potentially skewing results. Future studies should compare athletes and non-athletes or stratify samples by sport type, training intensity, and demographics to provide more nuanced insights. Given the significant role of saliva in oral health, further research could provide additional insights into the mechanisms involved in athletes’ oral health. Also, the results shall be interpreted with caution considering the observational design that precludes causal-effect interpretations, and for these longitudinal studies shall be conducted to further confirm whether there is causality in these associations.

## 5. Conclusions

The prevalence of dental caries and dental erosion was elevated in this cohort of athletes. Dietary patterns and oral hygiene habits varied and showed significant associations with measures of dental caries and dental erosion. These findings highlight the need for targeted dietary counseling and oral health education among athletes, whose nutritional demands may predispose them to higher consumption of sugar-rich diets.

## Figures and Tables

**Table 1:** Characteristics of participants.

Variable	Result
**Age, mean (SD)**	24.02 (4)
**Females, % (n)**	30.0 (24)
**Meals per day, % (n)**	
2–3 meals	53.8 (43)
4–5 meals	45.0 (36)
Don’t know	1.3 (1)
**Number of sports practiced, % (n)**	
1	77.5 (62)
2	15.0 (12)
3 or more	7.5 (6)
**Trains/week, median (IQR)**	4 (3)
**Hours/training, median (IQR)**	2.0 (0.5)
**Years practicing, % (n)**	
<5 y	77.5 (62)
5–10 y	22.5 (18)
10–15 y	30.0 (24)
15+ y	43.8 (35)
**Frequency of drinking sugary drinks or foods, % (n)**	
Never	3.8 (3)
Occasionally	53.8 (43)
Most days	36.3 (29)
All days	6.3 (5)

**Table 2:** Oral health habits, dental caries and dental erosion.

Variable	Result
**Oral hygiene habits, % (n)**	
Toothbrush	100.0 (80)
Flossing	53.8 (43)
Mouthwash	41.3 (33)
Interdental Brush	7.5 (6)
Tongue brush	11.3 (9)
Other	1.3 (1)
**Toothpaste with fluoride, % (n)**	67.5 (54)
**Regularity in dental visits, % (n)**	
Regularly, even without complaints	63.8 (51)
Only when in pain or with complaints	30.0 (24)
Never	2.5 (2)
Don’t know	3.8 (3)
**Last dental visit, % (n)**	
<6 months	60.0 (48)
6–12 months	23.8 (19)
>12 months	13.8 (11)
Don’t know	2.5 (2)
**My oral status has affected my training, % (n)**	15.0 (12)
**My oral status affected my competition, % (n)**	12.5 (10)
**Oral health self-assessment, % (n)**	
Satisfactory	20.0 (16)
Good	51.3 (41)
Very good	23.8 (19)
Bad	5.0 (4)
**Prevalence of caries lesions, % (n)**	50.0 (40)
**Number of caries lesions**	1.6 (2.6)
**Prevalence of erosion**	40.0 (32)
**BEWE overall score, mean (SD)**	1.9 (3.7)
**Erosion risk, n (%)**	
No risk	80.0 (64)
Mild	12.5 (10)
Moderate	5.0 (4)
High	2.5 (2)

**Table 3:** Consumption of different types of drink per participant.

	Never	1/Week	2–4/Week	4–6/Week	1/Day	2/Day	>2/Day
**Coffee**	28.8 (23)	8.8 (7)	20.0 (16)	17.5 (14)	1.3 (1)	7.5 (6)	47.5 (38)
**Tea**	48.8 (39)	23.8 (19)	13.8 (11)	8.8 (7)	0.0 (0)	1.3 (1)	58.8 (47)
**Carbonated drink**	22.5 (18)	31.3 (25)	33.8 (27)	11.3 (9)	0.0 (0)	0.0 (0)	35.0 (28)
**Lemonade**	22.5 (18)	25.0 (20)	22.5 (18)	26.3 (21)	2.5 (2)	0.0 (0)	50.0 (40)
**Energy drinks**	67.5 (54)	20.0 (16)	11.3 (9)	1.3 (1)	0.0 (0)	0.0 (0)	70.0 (56)
**Isotonic drinks**	46.3 (37)	27.5 (22)	18.8 (15)	5.0 (4)	1.3 (1)	1,3 (1)	52.5 (42)
**Isotonic gels**	68.8 (55)	17.5 (14)	13.8 (11)	0.0 (0)	0.0 (0)	0.0 (0)	70.0 (56)

**Table 4:** Multivariable linear regression for the number of dental caries.

	Adjusted—Model 1			Adjusted—Model 2		
Variable	Estimate	95% CI	*p*-Value	Estimate	95% CI	*p*-Value
(Intercept)	10.33	[1.06, 19.60]	0.033	8.98	[5.89, 12.07]	<0.001
Age	−0.03	[−0.20, 0.14]	0.737	-	-	-
Male	−0.39	[−1.99, 1.21]	0.635	-	-	-
BEWE	0.14	[−0.20, 0.49]	0.418	-	-	-
Flossing	0.11	[−1.26, 1.48]	0.874	-	-	-
4–5 meals per day	0.10	[−1.41, 1.62]	0.896	-	-	-
Don’t know how many meals per day	−1.30	[−10.28, 7.67]	0.777	-	-	-
Toothbrushing 2x/day	−0.21	[−2.18, 1.77]	0.837	-	-	-
Visit dentist—Never	−0.86	[−6.74, 5.02]	0.775	-	-	-
Visit dentist—Only when in pain or with complaints	−0.72	[−4.51, 3.08]	0.713	-	-	-
Visit dentist—Regularly, even without complaints	−0.82	[−4.56, 2.92]	0.668	-	-	-
Last dental visit—>12 months	−0.07	[−2.06, 1.91]	0.942	-	-	-
Last dental visit—6–12 months	0.73	[−1.08, 2.54]	0.431	-	-	-
Last dental visit—Don’t know	0.42	[−5.35, 6.18]	0.888	-	-	-
Oral Health Assessment—Good	−4.61	[−7.82, −1.40]	0.007	−5.01	[−7.39, −2.64]	<0.001
Oral Health Assessment—Satisfactory	−4.68	[−8.21, −1.16]	0.012	−5.21	[−7.75, −2.66]	<0.001
Oral Health Assessment—Very good	−4.82	[−8.39, −1.25]	0.011	−5.46	[−7.96, −2.95]	<0.001
Number of sports practiced	0.03	[−0.83, 0.89]	0.950	-	-	-
‘Trains/week’	0.10	[−0.37, 0.56]	0.683	-	-	-
Years practicing						
5–10 y	−0.22	[−4.42, 3.99]	0.919	-	-	-
10–15 y	−0.93	[−4.97, 3.12]	0.656	-	-	-
15+ y	−0.79	[−4.97, 3.40]	0.714	-	-	-
Frequency of drinking sugary drinks or foods, % (n)						
Never	−3.64	[−8.04, 0.76]	0.110	−1.94	[−4.15, 0.26]	0.089
Occasionally	−2.96	[−5.49, −0.42]	0.026	−3.60	[−6.95, −0.25]	0.038
Most days	−1.98	[−4.65, 0.69]	0.152	−3.02	[−5.18, −0.86]	0.008

**Table 5:** Adjusted multivariable linear regression for linear risk for dental erosion (BEWE as continuous variable).

	Adjusted—Model 1			Adjusted—Model 2		
Variable	Estimate	95% CI	*p*-Value	Estimate	95% CI	*p*-Value
(Intercept)	3.96	[−8.25, 16.17]	0.528	7.59	[3.88, 11.30]	<0.001
Age	0.10	[−0.13, 0.33]	0.412	-	-	-
Male	0.12	[−1.95, 2.19]	0.910	-	-	-
Caries (n)	0.14	[−0.20, 0.49]	0.418	-	-	-
4–5 meals per day	−0.54	[−2.60, 1.53]	0.611	−1.13	[−2.73, 0.46]	0.169
Don’t know how many meals per day	−13.01	[−25.04, −0.97]	0.038	−12.56	[−22.36, −2.75]	0.014
Toothbrushing 2x/day	0.73	[−1.98, 3.45]	0.598	-	-	-
Visit dentist—Never	3.33	[−4.21, 10.86]	0.390	-	-	-
Visit dentist—Only when in pain or with complaints	2.55	[−2.59, 7.69]	0.335	-	-	-
Visit dentist—Regularly, even without complaints	2.33	[−2.70, 7.35]	0.368	-	-	-
Last dental visit—>12 months	0.58	[−2.15, 3.32]	0.678	0.20	[−2.10, 2.51]	0.864
Last dental visit—6–12 months	−1.68	[−4.13, 0.76]	0.182	−1.64	[−3.66, 0.38]	0.116
Last dental visit—Don’t know	8.16	[0.51, 15.81]	0.041	8.82	[1.96, 15.68]	0.014
Oral Health Assessment—Good	−5.03	[−9.49, −0.57]	0.031	−5.28	[−8.82, −1.73]	0.005
Oral Health Assessment—Satisfactory	−3.85	[−8.66, 0.96]	0.122	−3.85	[−7.66, −0.04]	0.052
Oral Health Assessment—Very good	−6.01	[−10.84, −1.18]	0.018	−6.12	[−9.98, −2.27]	0.003
Number of sports practiced	−0.20	[−1.38, 0.98]	0.745	-	-	-
‘Trains/week’	−0.35	[−0.98, 0.29]	0.288	-	-	-
Years practicing						
5–10 y	−1.58	[−7.31, 4.14]	0.590	-	-	-
10–15 y	−0.54	[−6.25, 5.17]	0.853	-	-	-
15+ y	−0.45	[−6.03, 5.14]	0.876	-	-	-

## Data Availability

Data are fully available here https://doi.org/10.5281/zenodo.14622450.

## Supplementary Materials

The following supporting information can be downloaded at: https://www.mdpi.com/article/10.3390/nu17030543/s1, Table S1: STROBE statement—checklist of items that should be included in reports of observational studies; Table S2: multivariable linear regressions for dichotomic risk for dental caries; Table S3: multivariable linear regressions for dichotomic risk for dental wear.
